# Functional characterization of the matrix metalloproteinase-1 cigarette smoke-responsive region and association with the lung health study

**DOI:** 10.1186/1465-9921-13-79

**Published:** 2012-09-19

**Authors:** Alison M Wallace, Becky A Mercer, Jianqing He, Robert F Foronjy, Domenico Accili, Andrew J Sandford, Peter D Paré, Jeanine M D’Armiento

**Affiliations:** 1 University of British Columbia James Hogg Research Centre, St. Paul’s Hospital, Vancouver, BC, Canada; 2 Department of Medicine, Division of Molecular and Pulmonary Medicine, Columbia University College of Physicians and Surgeons, New York, NY, USA; 3 Department of Medicine, Division of Pulmonary and Critical Care Medicine, St. Luke’s Roosevelt Health Sciences Center, New York, NY, USA; 4 Naomi Berrie Diabetes Center and Department of Medicine, Columbia University, New York, New York, USA; 5 Department of Medicine, Division of Respiratory Medicine, University of British Columbia, Vancouver, BC, Canada

**Keywords:** Chromatin immunoprecipitation, COPD, Metalloproteinase, Polymorphisms, Transcription factors

## Abstract

**Background:**

Prior studies have demonstrated that the distal 1.5 kb of the MMP-1 promoter is fundamental in directing the induction of the MMP-1 gene by cigarette smoke.

**Methods:**

To characterize the genetic variants in the MMP-1 cigarette smoke-responsive element, deep re-sequencing of this element was performed on DNA samples from participants in the Lung Health Study. Furthermore, evidence of Sp1 binding to the MMP-1 promoter was assessed using chromatin immunoprecipitation assays and the influence of cigarette smoke exposure on this interaction was evaluated in cultured human small airway epithelial cells.

**Results:**

Ten polymorphisms (four novel) were detected in the cigarette smoke-responsive element. Chromatin immunoprecipitation assays to assess the protein-DNA interactions at Sp1 sites in the MMP-1 promoter showed increased binding to the Sp1 sites in the cigarette smoke-responsive element in small airway epithelial cells treated with cigarette smoke extract. In contrast, a Sp1 site outside of the element exhibited the opposite effect. None of the polymorphisms were more prevalent in the fast decliners versus the slow decliners (fast decliners = mean −4.14% decline in FEV1% predicted per year vs. decline in FEV1% predicted per year).

**Conclusions:**

Sequencing analyses identified four novel polymorphisms within the cigarette smoke-responsive element of the MMP-1 promoter. This study identifies functional activity within the cigarette smoke-responsive element that is influenced by cigarette smoke and examines this region of the promoter within a small patient population.

## Background

Chronic obstructive pulmonary disease (COPD), which is characterized by both emphysema and inflammatory scarring and narrowing of small airways, is a major cause of morbidity and mortality worldwide [1]. Cigarette smoke is the single most important factor in the development of COPD. Not all smokers develop COPD, however, smoking is responsible for up to 90% of cases in the developed world [2]. Current diagnostic and therapeutic options for this disease are limited.

Much attention has been given to the role of matrix metalloproteinases (MMPs), a family of zinc-dependent proteinases with the capacity to degrade both elastin and collagen, in the pathogenesis of COPD. Although the original protease-antiprotease imbalance theory of COPD focused on destruction of elastin in the lung, there is evidence that collagen degradation is important as well. In 1992 D’Armiento and coworkers [3] found that over-expression of human MMP-1 (interstitial collagenase) in transgenic mice led to the development of emphysema. Subsequent studies demonstrated that the important target for MMP-1 was type III collagen and that adult-onset emphysema developed in strains of mice expressing MMP-1 in the lung [3,4]. In humans, increased levels of MMP-1, localized within the resident alveolar epithelial cells, have been reported in the lungs of patients with emphysema but not in normal controls [5,6]. In addition, Type II pneumocytes are also known to express MMP-1 [5]. *In vitro*, direct exposure of human small airway epithelial cells to cigarette smoke induced MMP-1 mRNA and protein expression via the extracellular-signal-regulated kinases (ERK)/mitogen activated protein kinase (MAPK) pathway and human emphysematous lung tissue showed significantly increased ERK activity compared to control lung [7]. Through deletion studies our laboratory identified that the distal 1.5 kb of the MMP-1 promoter was fundamental to the direct induction of the MMP-1 gene by cigarette smoke and that Sp1 is activated by cigarette smoke [8]. 

Only a small subset of smokers develop COPD and once established, the disease process varies greatly from person to person. There is an underlying genetic susceptibility determining both the onset and the course of the disease.The heterogeneity of this disease suggests that there may be some genes and/or gene variants that contribute to COPD in general, while other genes and/or gene variants may be relevant only for a particular phenotype. It is unknown whether DNA sequence variation in the MMP-1 gene contributes to the individual susceptibility and/or phenotypic variation. We hypothesized that DNA sequence variation in this responsive element could be associated with a COPD phenotype, accelerated lung function decline, and if an association was found may suggest a functional role of these polymorphisms. Furthermore, based on our previous work, we speculated that gene variants could modulate Sp1 binding and therefore influence smokers’ susceptibility to COPD through variations in MMP-1 expression.

In the present study the genetic variants in the cigarette smoke-responsive region were characterized, with particular interest in identifying single nucleotide polymorphisms (SNPs) altering Sp1 binding sequences. Chromatin immunoprecipitation (ChIP) assays were performed to assess the protein-DNA interactions at Sp1 sites in the MMP-1 promoter to define the functional binding sites and determine whether or not these sites were influenced by cigarette smoke exposure in cultured human small airway epithelial cells. An association study was also performed to test whether SNPs within this region were associated with an accelerated rate of decline in lung function in the Lung Health Study (LHS) participants.

## Methods

### Study population

DNA for re-sequencing and genetic association studies was obtained from subjects selected from participants in Phase I of the National Heart, Lung, and Blood Institute LHS. Details of the study have been previously published [9]. Briefly, study participants were current smokers, 35–60 years of age, who had mild to moderate airflow obstruction (FEV1 55–90% predicted and FEV1/FVC ≤ 0.70). The primary outcome variable was rate of decline in post-bronchodilator FEV1 over a follow-up period of five-years. A subset of 582 individuals were selected from the 5,887 subjects in the LHS because they represent phenotypic extremes. They include the 277 subjects (fast decliners) who continued to smoke during the first five-years of follow-up, had yearly measurements of FEV1, and showed the fastest decline in lung function over that time (FEV1% predicted change = −4.14%/year), and the 305 subjects (slow decliners) who continued to smoke and exhibited the slowest decline over the same period of time (FEV1% predicted change = +1.07%/year). Of the subset, 551 non-Hispanic white participants (261 fast decliners and 290 slow decliners) were selected for the association study. This study was approved by the University of British Columbia/Providence Health Care Research Ethics Board.

### Identification of DNA sequence variants

High-throughput sequencing of the MMP-1 cigarette smoke-responsive element (~1.5 kb) was performed at the Genome Sciences Centre at the British Columbia Cancer Agency using Applied Biosystems chemistries (ABI BigDye™ v3.1 Terminator Chemistry) and equipment (ABI 3730xl). Sequencing PCR primers were designed using Overlapping Primersets ( http://www2.eur.nl/fgg/kgen/primer/Overlapping_Primers.html). The primer sequences were: Region 1 (−3660 to −4343) sense 5’-CTAAAAGCTCTGCAGGCCAC-3’ and antisense 5’-CGCTTAGGCTGGAGTGTAGG-3’ region 2 (−3510 to −4147) sense 5’-GGCATGCAAATCACCAAAA-3’ and antisense 5’-AATCCTCCCCTTCAAGCTGT-3’ region 3 (−2890 to −3748) sense 5’-TCTTAGGAGAGTAAAAGTCATGGACA-3’ and antisense 5’-TTCTTGGTTGCTTCATGCTG-3’. For the purpose of sequencing, a M13 forward linker was added to the 5’ end of the forward primers (TGTAAAACGACGGCCAGT) and a M13 reverse linker was added to the 5’ end of the reverse primers (CAGGAAACAGCTATGAC). Sequencing was performed using M13 universal sequencing primers. PCR conditions were as follows: initial denaturation at 95°C for 15 minutes; 35 cycles at 94°C for 30 seconds, 58°C for 30 seconds, and 72°C for 50 seconds; and final extension at 72°C for 10 minutes. Discovery of sequence variation was performed using novoSNP [10], which has higher sensitivity and specificity than PolyPhred [11]. Each SNP had a completion rate of greater than 90% in all subjects and a Hardy-Weinberg equilibrium *P* value of greater than 0.01 in non-Hispanic whites.

### Chromatin immunoprecipitation

ChIP was performed using the Upstate (Millipore) ChIP Kit (Billerica, MA, USA) according to the manufacturer’s instructions. Chromatin was harvested from human small airway epithelial cells (Lonza, Walkersville, MD) that were seeded at 7.5×10^5^ cells per T75 flask (BD Falcon, San Jose, CA) and treated with control media or 5% cigarette smoke extract at 80% confluence for 24 hours, prior to crosslinking, as previously described [7]. Cells between passages two and six were used in all *in vitro* experiments. Conditions were established to obtain chromatin fragments 200–600 bp in length using the Fisher Scientific F 550 Sonic Dismembrator. Immunoselection of chromatin fragments was performed using 2 ug of rabbit polyclonal Sp1 antibody (sc-59 X; Santa Cruz Biotechnology, Santa Cruz, CA). An aliquot that was immunoprecipitated without antibody was used as a negative control. Detection of specific DNA sequences was performed using real-time quantitative PCR with the use of an ABI Prism 7900HT Sequence Detection System (Applera Corporation, Norwalk, CT, USA) using the following primers: -3987 site (83 bp amplicon) sense 5’-TCTCCAGTAAGGCTGGGTGT-3’ and antisense 5’-CTGGCCTCAAGCAGTTCTCT-3’;-3455 site (117 bp amplicon) sense 5’-TGCAGACACCTACTATGTTGAG-3’ and antisense 5’-ATAATGTCACCATGCCACCAC-3’; and-2209 site (68 bp amplicon) sense 5’-TAGAGAAGGGAGGAAAAAGCAG-3’ and antisense 5’-GTTGGAAATAGAGCCTTGGAGT-3’.

### Statistical analysis

The associations were analyzed by binary logistic regression to adjust for potential confounding factors. The outcome was a dichotomous variable, that is, fast decline or slow decline. Potential confounding factors included in the analysis were age, sex, smoking history (expressed as pack-years), initial level of lung function (pre-bronchodilator FEV1 percent predicted), and methacholine responsiveness. The latter variable was expressed as a two-point dose–response slope as previously described [12]. Hardy–Weinberg equilibrium was calculated using an online calculator ( http://www.oege.org/software/hwe-mr-calc.shtml) [13]. Minimal detectable odds ratio at *P* = 0.05 and 0.80 power for different minor allele frequency (MAF) in the case–control association study was performed using PGA Power Calculator software [14]. All other tests were performed using the JMP Statistics software package (SAS Institute Inc., Cary, NC). Statistical significance was defined at the 5% level.

## Results

Descriptive statistics and univariate comparisons between fast decliners and slow decliners are summarized in Table 1. Age, sex, smoking history (pack-years), baseline FEV1% predicted postbronchodilator, and methacholine response were borderline or significantly different between the two groups. Therefore, the association of genotypes with decline of lung function was analyzed by logistic regression to adjust for these factors.

**Table 1:** Characteristics of study subjects

	**Slow decliners (n = 290)**	**Fast decliners (n = 261)**	***P *value**
Age	47.4 ± 0.4	49.6 ± 0.4	0.0007
Male (%)	193 (66.6)	155 (59.4)	0.082
Pack-years	38.3 ± 1.1	43.2 ± 1.2	0.002
Baseline FEV1, % predicted*	79.8 ± 0.5	74.6 ± 0.6	<0.0001
Methacholine response†	−8.0 ± 0.9	−24.2 ± 2.1	<0.0001

Data are presented as percent or mean (SE).

*Lung function at the start of the study as measured percent predicted FEV1 (postbronchodilator).

†Measurement of the responsiveness of the airways to methacholine expressed as percent decline in FEV1 per final cumulative dose of methacholine administered (O'Connor et al. 1995).

Re-sequencing of the MMP-1 cigarette smoke-responsive region revealed a total of ten polymorphisms (Table 2). Four of these genetic variants have not been previously reported in the National Center for Biotechnology Information SNP database (dbSNP). The presence and frequency of six dbSNPs were confirmed; three previously reported dbSNPs were not identified.

**Table 2:** Identification of polymorphisms in MMP-1 cigarette smoke-responsive element

SNP*	**SNP ID**	**WW†**	**WM†**	**MM†**	**NC‡**	**Not amplified for this SNP**	**Total N**	**Failed %**	**W%**	**M%**
17 T/A	rs484915	167	265	126	4	20	582	4.1	53.7	46.3
352 G/A	rs470307	554	9	0	0	19	582	3.3	99.2	0.8
412 T/A	NA	571	5	0	0	6	582	1.0	99.6	0.4
712 G/A	rs2408490^§^	368	149	16	4	45	582	8.4	83.0	17.0
724 T/A	rs12279710	534	3	0	1	44	582	7.7	99.7	0.3
759 T/G	rs7107224	391	162	19	5	5	582	1.7	82.5	17.5
816A/C	rs1155764^§^	383	157	21	19	2	582	3.6	82.3	17.7
1164C/T	rs34695796	537	19	1	9	16	582	4.3	98.1	1.9
1227 G/A	NA	558	6	0	2	16	582	3.1	99.5	0.5
1283 G/A	NA	554	5	0	7	16	582	4.0	99.6	0.4
1284 G/A	NA	499	49	3	15	16	582	5.3	95.0	5.0

* Position in AF023338.

† “W” denotes wild-type allele; “M” denotes mutant allele.

‡Results are not consistent between forward primer sequencing and reverse primer sequencing.

§Rs2408490 and rs1155764 are in complete LD with r^2^ = 1 in Hapmap database.

Several transcription factors are induced by cigarette smoke and have been shown to be important for regulating MMP-1 expression [8]. Since not all smokers develop COPD, despite smoking being the most important risk factor for the development of this disease, gene variants in the cigarette smoke-responsive element could provide a susceptibility locus for smoke-induced lung disease. None of the polymorphisms in this region correspond specifically to the Sp1 sites at positions −3987 and −3455 that through site-directed mutagenesis we know are important for regulating MMP-1 expression (data not shown). Furthermore, other transcription factor binding sites of interest, such as AP-1 sites at positions −4293 and −4210 and c-Ets-1 sites at positions −3838 and −3344, did not overlap specifically with any polymorphisms identified in this cohort of smokers, possibly suggesting that the transcription factor binding sites are highly conserved. Despite not directly altering the above mentioned binding sites, these polymorphisms could affect the binding of other regulatory proteins that control the activity of the promoter and influence mRNA and protein levels.

**Figure 1:**
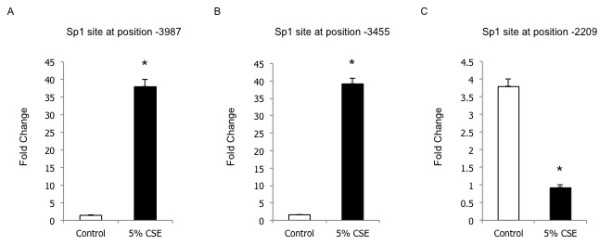
** Sp1 action on the MMP-1 promoter is shown by the fold change in specific DNA sequences detected by qPCR.** MMP-1 ChIP assays were done in cell cultures from human small airway epithelial cells with and without cigarette smoke extract (CSE) treatment using an antibody to Sp1 or nonimmune serum. We amplified DNA using primers spanning two Sp1 binding sites within the cigarette smoke-responsive element (**a**,**b**) and one sequence outside of this element (**c**). Samples from each experiment were run in triplicate. Data are mean ± SEM. *P < 0.05.

Our previous studies have shown that in lung epithelial cells Sp1 modifies the expression of MMP-1 during smoke exposure [8]. Therefore, we next investigated the mechanism by which Sp1 regulates MMP-1 expression. To test the hypothesis that Sp1 is a key regulator of MMP-1 expression in intact cells, we performed ChIP assays on human small airway epithelial cells, *in vitro*. The DNA corresponding to the Sp1 sites in the cigarette smoke-responsive element was amplified and chromatin immunoprecipitated with an antibody to Sp1. The data demonstrated that Sp1 is recruited onto the MMP-1 promoter *in vitro*. The results show an interaction between Sp1 and the MMP-1 promoter at baseline and that there is a significant increase in binding in cultured human small airway epithelial cells treated with cigarette smoke extract (Figure 1a-b). In contrast, the Sp1 site at position −2209, which is outside of the cigarette smoke-responsive element, exhibited the opposite effect (Figure 1c). 

**Figure 2:**
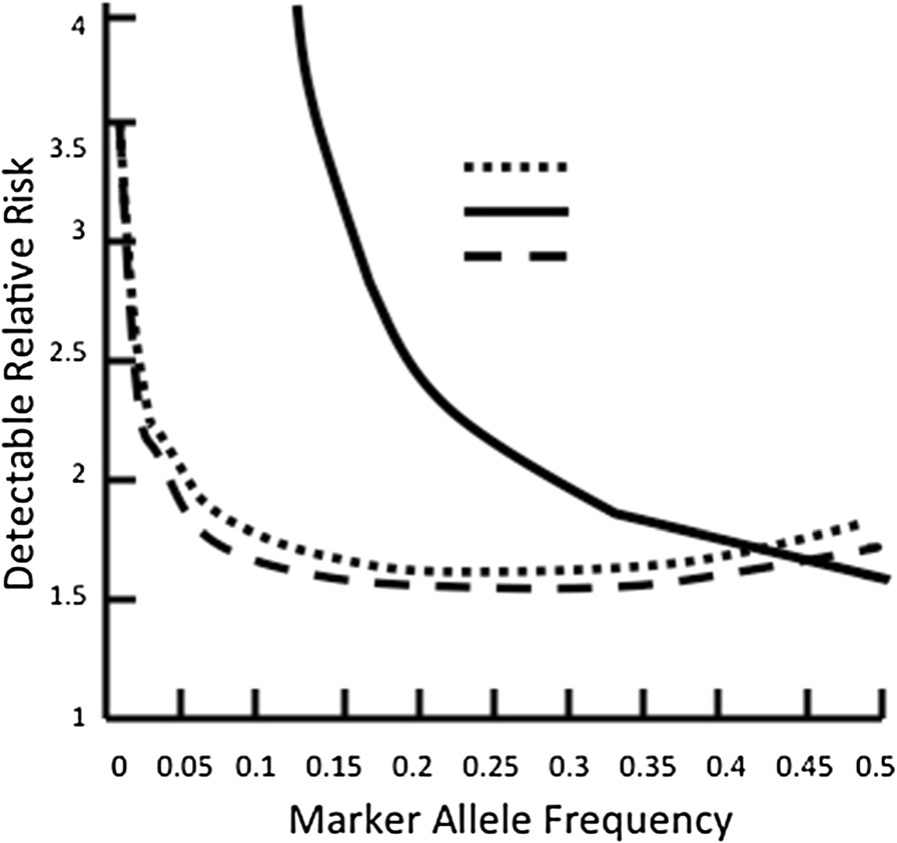
The detectable relative risk given the number of subjects is shown for a variety of marker allele frequencies using different genetic models.

Association analyses of the individual SNPs with rate of decline in lung function in the fast decliners and slow decliners showed no significant associations between genetic variants in the MMP-1 cigarette smoke-responsive element and this COPD phenotype in non-Hispanic whites (Table 3). Minimal detectable odds ratios for different MAFs and different genetic models are shown in Figure 2. The study design has 80% power to detect associations with odds ratios of 2.0 for the five SNPs with a MAF ≥ 0.05 in co-dominant and dominant genetic models.

**Table 3:** Association with rate of decline of lung function non-Hispanic whites

**SNP position in AF023338**	**Rs in dbSNP**	**Chromosome 11 position**	**Slow decliners**			**Fast decliners**			***P *value**
			**WW**	**WM**	**MM**	**WW**	**WM**	**MM**	
17 T/A	rs484915	102178458	73 (26%)	136 (49%)	70 (25%)	78 (31%)	117 (47%)	53 (21%)	0.35
352 G/A	rs470307	102178123	274 (98%)	7 (2%)	0	249 (99%)	2 (1%)	0	0.18
412 T/A	NA	102178063	283 (99%)	3 (1%)	0	257 (99%)	2 (1%)	0	1.00
712 G/A	rs2408490	102177763	191 (72%)	64 (24%)	9 (3%)	162 (67%)	73 (30%)	6 (2%)	0.28
759 T/G	rs7107224	102177716	200 (71%)	72 (25%)	11 (4%)	175 (68%)	77 (30%)	7 (3%)	0.44
816 A/C	rs1155764	102177659	196 (71%)	69 (25%)	11 (4%)	171 (67%)	76 (30%)	9 (3%)	0.47
1164 C/T	rs34695796	102177311	263 (96%)	12 (4%)	0	244 (97%)	7 (3%)	1 (0%)	0.36
1227 G/A	NA	102177248	277 (99%)	3 (1%)	0	251 (99%)	3 (1%)	0	1.00
1283 G/A	NA	102177192	274 (99%)	3 (1%)	0	251 (99%)	2 (1%)	0	1.00
1284 G/A	NA	102177191	247 (91%)	23 (8%)	2 (1%)	225 (90%)	25 (10%)	1 (0%)	0.74

## Discussion

MMP-1 is important in the pathogenesis of COPD with increased expression being a common finding in patients with this disease [5,6,15]. Animal studies have shown that MMP-1 plays a key role in the initiation of the disease process [3]. Previous studies from our laboratory have identified the distal MMP-1 promoter to be fundamental for the direct activation of the MMP-1 promoter by cigarette smoke [8]. Studies of genetic variants in the MMP-1 promoter are important because identification of functional polymorphisms will lead to a greater understanding of the regulatory mechanisms involved in both health and disease, and may provide useful knowledge for identifying at-risk individuals to allow for early interventions.

This present study generated the following three important findings. First, the genetic variants in the MMP-1 cigarette smoke-responsive element were further characterized and four novel polymorphisms identified, as well as the confirmation of six previously reported genetic variants. Second, an important interaction between Sp1 binding and the MMP-1 cigarette smoke-responsive element in human small airway epithelial cells exposed to cigarette smoke extract was documented. Sp1 protein binding was influenced by cigarette smoke and this DNA-protein interaction occurred specifically in the cigarette-smoke responsive element and did not appear to occur outside of the core regulatory region. Third, polymorphisms in the cigarette smoke-responsive element were not more prevalent in subjects who had an accelerated decline in lung function over five-years of the Lung Health Study.

MMP-1 is tightly regulated at the level of transcription, post-transcription, and post-translation; it is also known that MMP-1 expression is influenced by genetic variants in the promoter [16]. In the cigarette smoke-responsive element spanning approximately 1.5 kb, a total of ten polymorphisms were identified via re-sequencing. Four novel polymorphisms were revealed. These data are important because they allow researchers to further analyze the contribution of genetic polymorphisms to the development and progression of multiple diseases associated with MMP-1 expression. In addition to the established role of MMP-1 in COPD, elevated expression of MMP-1 has been associated with a variety of pathological conditions such as rheumatoid and osteoarthritis, atherosclerosis, Alzheimer disease, and cancer [17]. There is a functional polymorphism in the MMP-1 promoter (rs1799750) described by Rutter et al. that has been associated with numerous malignant processes [18]. Further understanding of the genetic variants in the MMP-1 promoter, and more specifically the cigarette smoke-responsive element, may provide additional insights into the molecular mechanisms of these diseases and help define the role of smoking as a risk factor.

Understanding the molecular mechanisms controlling MMP-1 expression may identify potential targets for the prevention of diseases and degenerative conditions associated with dysregulation of MMP-1. The first cis-promoter element found to be responsible for regulating MMP-1 gene expression was AP-1 [19]. Previous studies by our laboratory have identified a number of transcription factors, including AP-1, that are important for regulating MMP-1 promoter activity [8]. Further analysis in the present study revealed two Sp1 sites (−3987 and −3455) in the cigarette smoke-responsive element that have now been analyzed using a ChIP assay to assess the functional importance of these sites *in vitro*. Our data demonstrates that these are functional binding sites and that binding of Sp1 to the MMP-1 promoter is influenced by cigarette smoke. This is in contrast to the decreased binding of Sp1 to the MMP-1 promoter outside of the cigarette smoke responsive-element in response to cigarette smoke exposure. Not only are these sites functional, they also occur in a region harbouring a number of common SNPs. Although the genetic variants identified did not directly alter the primary transcription factor binding sites we assessed, they may influence the binding of chromatin modifying proteins resulting in a measurable functional effect on promoter activity. Unfortunately, this study is limited by the fact that susceptibility to COPD (i.e. accelerated decline in lung function with smoking) and Sp1 binding to the MMP-1 gene were not measured in the same individuals since the appropriate cells were not available from the LHS participants. However, the findings that the SNPs identified do not directly alter the Sp1 bindings sites at the DNA level and are not associated with rapid decline in lung function are interesting and potentially important. It may be that since Sp1 plays a significant role regulating MMP-1 expression these sites are conserved. These negative results do suggest that the SNPs are not a risk for rapid decline in lung function among smokers.

COPD is a chronic disease of complex phenotype [20,21]. There is even speculation that COPD represents a syndrome comprised of many rare diseases [22]. In the present association study the relationship between polymorphisms in the cigarette smoke-responsive element and one COPD phenotype, rate of decline in lung function, was tested. No significant associations were found despite the sufficient power of the study. Such a finding however is not surprising since the results of the basic studies suggest that MMP-1 expression is critical in the pathogenesis of lung destruction and generation of the emphysema phenotype [3]. It will be necessary in future detailed human studies to test for an association of the cigarette smoke-responsive element polymorphisms with CT evidence of lung destruction. Unfortunately, CT data was not available on the LHS participants and therefore we were unable to investigate this further in our population. Additionally, our lab has demonstrated that cigarette smoke directly induces the expression of MMP-1 highlighting the importance of MMP-1 in the susceptibility to lung destruction in smokers [3]. All LHS participants exhibit some degree of airflow limitation, indicating that they are already responsive to the damaging effects of cigarette smoke. Therefore, to test the importance of this element in disease susceptibility it will be necessary to assess these genetic variants in alternative well-characterized cohorts of smokers with and without COPD, as well as normal non-smokers.

We acknowledge several limitations to the present study in addition to the issues outlined above. Although an association between the MMP-1 polymorphisms and rate of decline in lung function was not identified, these polymorphisms may influence other COPD-related phenotypes. We know that lung function normally declines as part of the aging process, while accelerated lung function decline is a hallmark of COPD [23,24], therefore, the use of this phenotype seems reasonable for genetic association studies and decline in lung function over time has been a common end point in clinical trials of COPD therapies [25,26]. However despite the rigor of the lung function phenotyping in the LHS, the five-year period of follow-up may not be sufficient to clearly separate groups whose decline in lung function varied significantly over time. Indeed, the use of this single COPD phenotype and a short follow-up period are limitations of this study. On the other hand our group has previously identified susceptibility variants in candidate genes using this approach [27,28]. The association study was limited to non-Hispanic white subjects but the genetic basis of COPD may differ between racial/ethnic groups as several COPD association studies have shown distinct results within different races. Indeed, it is known that MMP-1 polymorphisms are associated with diseases such as cancer in other ethnic groups [29,30]. Therefore, it will be of interest to further investigate the role of these polymorphisms in alternative racial/ethnic populations. Future studies may also include haplotype analysis to fully characterize the genetic architecture of this region.

## Conclusions

In conclusion, the genetic variants in the MMP-1 cigarette smoke-responsive element have been further characterized revealing four novel polymorphisms. Evidence has been generated providing insight into the transcriptional regulation of the MMP-1 promoter in response to cigarette smoke exposure, which could have a significant impact on the understanding of many age-associated degenerative disease processes. COPD association studies are challenging since COPD is a heterogeneous condition and many phenotypes exist. Although a significant association between these MMP-1 polymorphisms and an accelerated rate of decline in lung function in subjects with some degree of airflow limitation was not identified, future studies performed on a cohort of smokers with and without disease needs to be carried out to determine if the SNPs in the MMP-1 promoter associate with disease susceptibility as would be predicted from the basic bench research. Furthermore, future studies are planned to further elucidate the mechanisms regulating MMP-1 expression and the functional consequences of this altered expression, which includes mapping out which transcription factor binding sites, if any, are altered by SNPs in this region and determining the functionality. As we found with Sp1, AP-1, and c-Ets-1 binding sites, we speculate that other transcription factor binding sites in this region may be highly conserved and therefore unaltered. Despite this, although not directly altering the binding of transcription factors, gene variants in this region could influence the multi-protein complexes that are formed during transcription and modulate MMP-1 expression.

## Competing interests

The authors declare that they have no competing interests.

## Authors’ contributions

AW participated in the study design, prepared the samples for sequencing, carried out the chromatin immunoprecipitation assays, participated in the statistical analysis, and drafted the manuscript. BM participated in the design of the study. JH performed the sequencing analysis and association studies. RF participated in the design of the study and helped draft the manuscript. DA provided his expertise with the chromatin immunoprecipitation assays. AS participated in the design of the study, participated in the statistical analysis, and helped draft the manuscript. PP and JD conceived of the study, participated in its design and coordination, and helped draft the manuscript. All authors read and approved the final manuscript.
